# Crossing the Widom Line: Evolution of Cohesive Strength and Cavity Formation in Supercritical Water

**DOI:** 10.1021/acs.jpclett.6c01190

**Published:** 2026-08-03

**Authors:** Jiacheng Niu, Qiuyang Zhao, Michael J. Adams, Hao Lu, Yin Chen, Xinjian Wang, Hui Jin, Liejin Guo

**Affiliations:** † State Key Laboratory of Multiphase Flow in Power Engineering, 12480 Xi’an Jiaotong University, Xi’an, Shaanxi 710049, People’s Republic of China; ‡ School of Chemical Engineering, 1724 University of Birmingham, Birmingham B15 2TT, United Kingdom

## Abstract

Supercritical water (SCW) is a promising green solvent, yet optimizing its performance requires a fundamental understanding of the thermodynamic behavior and solvation mechanisms. Here, molecular dynamics simulations are used to investigate the thermodynamic responses, cohesive-strength evolution, and cavity-formation behavior of SCW along isobars, with the NIST benchmark data serving as a reference for the classical Widom line (WL) behavior. The origin of the high-pressure WL divergence is analyzed from the perspective of statistical fluctuations and intermolecular interactions. Furthermore, cohesive energy density and the state-dependent mean-radius cavity-formation free energy, together with their corresponding thermal response coefficients, are introduced to establish a thermodynamic connection with the classical WL. Building on this connection, the high-pressure “Widom region” is delineated, and the thermodynamically driven evolution sequence along the isobaric heating path is revealed. This work provides a thermodynamic framework for understanding the evolution of the Widom region and its relevance to the solvation behavior of SCW.

## Graphical abstract

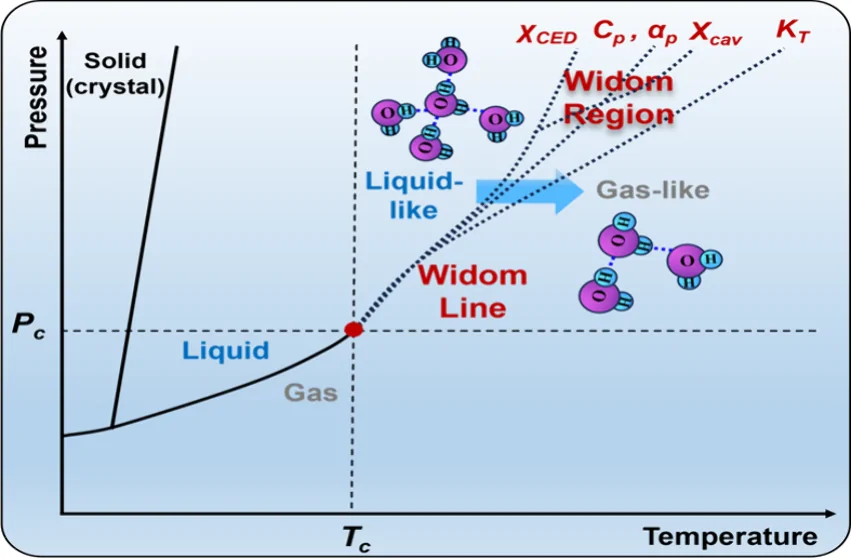

Supercritical water (SCW) exhibits both the high diffusivity of gases and the excellent solvation capacity of liquids,^1,2^ making it a promising green solvent and reaction medium for waste treatment,^3,4^ biomass conversion,^5,6^ materials synthesis,^7,8^ and deep geothermal power generation.^9^ However, the high energy consumption and severe equipment corrosion associated with extreme supercritical conditions hinder large-scale industrial applications.^10,11^ Optimizing the operating parameters to reduce energy consumption and boost conversion efficiency is therefore critical to promoting the deployment of this technology, a goal that fundamentally relies on a deep understanding of the evolution of the physicochemical properties of SCW. Among these properties, understanding the intrinsic mechanism of the solvation behavior remains a long-standing fundamental scientific challenge.^12^

In classical thermodynamics, supercritical fluids (SCFs) were originally described as being a homogeneous single phase.^13^ However, extensive studies have since revealed significant local inhomogeneities, where microscopic structure and macroscopic thermodynamic properties undergo dramatic and coupled changes.^14–16^ According to scaling theory, the correlation length (*ξ*) of internal fluctuations diverges as the system approaches the liquid–gas critical point (LGCP), leading to distinct maxima in the thermodynamic response functions including the isobaric heat capacity (*C*_*p*_), thermal expansion coefficient (α_*p*_), and isothermal compressibility (*K*_*T*_).^17^ These maxima converge to form the Widom line (WL)^18,19^ (Figure 1), the natural extension of the liquid–gas coexistence curve into the supercritical region, which separates the “liquid-like” and “gas-like” regimes through a dynamic crossover.^20,21^ When applied to SCW, this dynamic crossover manifests as a drastic transition in transport properties.^22,23^ Subsequently, the WL concept has been widely applied to understanding the physicochemical behavior of SCW.^24,25^ Notably, as greater pressures have been considered, the extrema trajectories of the different response functions fail to coincide, indicating a gradual transition zone bounded by multiple crossover lines rather than a single WL.^26^

**Figure 1:**
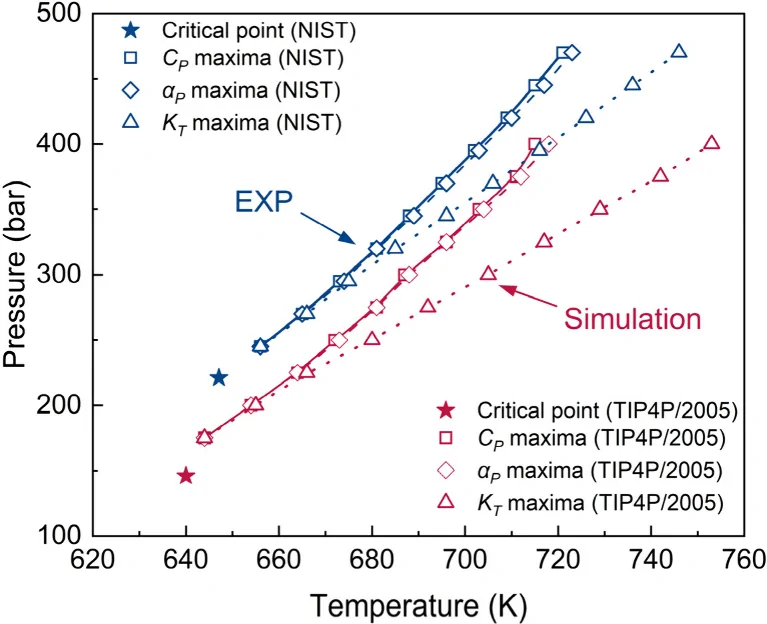
Widom lines in the *P–T* plane from the thermodynamic response functions (eqs 1–3) based on NIST benchmark data (blue) and TIP4P/2005 simulations (red). The trajectories correspond to the maximum values of *C*_*p*_ (squares, solid lines), α_*p*_ (diamonds, dashed lines), and *K*_*T*_ (triangles, dotted lines). Stars denote the vapor*–*liquid critical points. The curves were interpolated at 1 K intervals and smoothed via cubic spline interpolation. The agreement between the simulated trajectories and previously reported Widom line trajectories supports the reliability of the MD methodology used in the current work.^22,23^

Previous studies have offered valuable physical insights into this trajectory separation by linking it to microscopic structural changes, local density fluctuations, and dynamic crossovers.^16,22,27–30^ However, how different fluctuation contributions drive the high-pressure separation of these response maxima remains insufficiently clarified, limiting a thermodynamic understanding of the state evolution of SCW within the high-pressure region. Meanwhile, according to classical solution thermodynamics, the overall solvation process is governed by a complex balance of solute–solvent interactions, solvent–solvent cohesion, solute-induced solvent reorganization, and the accompanying entropic and pressure–volume contributions.^32,33^ Within this framework, the cohesive strength of the pure solvent is intrinsically linked to the cavity-formation penalty, which serves as a thermodynamic prerequisite for solvation.^34–36^ Although solvent cohesive strength and the associated cavity-formation penalty do not fully determine the overall solvation free energy, they provide important thermodynamic measures of the solvent contribution to solvation and have been widely discussed in the context of standard liquid solutions.^37–41^ Nevertheless, their connection to the WL and the supercritical crossover, as well as the evolution of this connection under high-pressure conditions, remains insufficiently established.

To address these issues, the present study employs molecular dynamics (MD) simulations using the TIP4P/2005 force field,^42^ with the NIST benchmark data^43^ as a reference for assessing the classical WL behavior. All fluctuation, cohesive-strength, and cavity-formation analyses are performed using the same MD data set to ensure internal consistency. First, the classical WL is defined from the maxima of the thermodynamic response functions, and the origin of its high-pressure divergence is revealed from the perspective of statistical fluctuations and intermolecular interactions. Subsequently, the cohesive energy density and its thermal response coefficient are introduced to describe the bulk evolution of solvent cohesive strength. In parallel, the state-dependent mean-radius cavity-formation free energy and its thermal response coefficient are employed to characterize the thermodynamic evolution of cavity formation. The resulting cohesive-response and cavity-response ridges identify the thermodynamic trajectories along which solvent-network cohesion attenuates most rapidly and the cavity-formation free energy decreases most rapidly, respectively. The relationship between these response ridges and the classical WL trajectories further provides a framework for delineating the high-pressure “Widom region” and characterizing the thermodynamically driven evolution sequence along the isobaric heating path.

To determine the thermodynamic trajectory of the WL in *P–T* space, the values of *C*_*p*_, α_*p*_ and *K*_*T*_ were calculated along isobaric paths from the TIP4P/2005 simulations, and the ridge lines of their maxima were identified accordingly. The NIST benchmark data were employed as a reference for comparison. These functions are defined by the following expressions:

1 $$C_{p}=\frac{1}{N}{\left(\frac{\partial H}{\partial T}\right)}_{P}=\frac{\left\langle {\left(\delta H\right)}^{2}\right\rangle }{k_{B}T^{2}}$$

2 $$\alpha_{p}=-\frac{1}{\rho }{\left(\frac{\partial \rho }{\partial T}\right)}_{P}=\frac{\left\langle \delta H\delta V\right\rangle }{k_{B}T^{2}V}$$

3 $$K_{T}=\frac{1}{\rho }{\left(\frac{\partial \rho }{\partial P}\right)}_{T}=\frac{\left\langle {\left(\delta V\right)}^{2}\right\rangle }{k_{B}TV}$$

where *H* is the enthalpy and *k*_B_ is the Boltzmann constant.

As shown in Figure 1, the TIP4P/2005 model accurately reproduces the trends of the thermodynamic maxima ridge lines obtained from the NIST benchmark data, which is consistent with the systematic results of Gallo et al.^22,23^ The maxima trajectories of *C*_*p*_ and α_*p*_ remain highly coincident across the supercritical region. By contrast, the *K*_*T*_ maxima ridge line in the TIP4P/2005 simulations follows these trajectories only in the near-critical region, within approximately 25 K and 80 bar above the model critical point, before deviating significantly at higher pressures.

Near the critical point, the system is dominated by macroscopic long-range density fluctuations (i.e., the divergence of ξ).^17^ The evolution of the thermodynamic response functions is strictly constrained by universal scaling laws, leading to highly synchronized behavior and near-perfect coincidence of the maxima ridge lines for *C*_*p*_, α_*p*_ and *K*_*T*_ in this regime. With increasing pressure, the average intermolecular distance decreases, free volume is significantly reduced, and short-range intermolecular repulsive interactions intensify sharply.^33^ Under such highly confined conditions, it becomes more difficult both to overcome local steric hindrance in order to achieve microscopic structural rearrangement, and to overcome the high external pressure for generating macroscopic density fluctuations. Consequently, as the pressure increases, the characteristic response peaks of *C*_*p*_, α_*p*_ and *K*_*T*_ are shifted to progressively higher temperatures. Notably, the high-temperature shift of the *K*_*T*_ maxima trajectory is significantly greater than those of *C*_*p*_ and α_*p*_ (Figure 1). To reveal the physical origin of this deviation, the microscopic fluctuation terms of the different response functions were further analyzed to clarify the statistical thermodynamic and intermolecular interaction mechanism driving the high-pressure divergence of these extremum trajectories.

The value of *C*_*p*_ reflects the enthalpy fluctuations ⟨(*δH*)^2^⟩ of the system. According to fluctuation theory, the enthalpy fluctuations can be decomposed into three contributions, as shown in eq 4:

4 $$\left\langle {\left(\delta H\right)}^{2}\right\rangle =\left\langle {\left(\delta U+P\delta V\right)}^{2}\right\rangle =\left\langle {\left(\delta U\right)}^{2}\right\rangle +P^{2}\left\langle {\left(\delta V\right)}^{2}\right\rangle +2P\left\langle \left(\delta U\delta V\right)\right\rangle$$

Based on eqs 1 and 4, *C*_*p*_ can be divided into three components.^44^ These are an internal energy self-fluctuation term (*C*_*p, U*_), a pressure–volume work self-fluctuation term (*C*_*p, PV*_), and an energy-volume cross-correlation term (*C*_*p, U–V*_). As shown in Figure 2a and b, the contribution of *C*_*p, PV*_ remains consistently small in both the near-critical low-pressure region at 200 bar and the high-pressure region at 400 bar, and the position of the *C*_*p*_ maximum closely coincides with the peak of *C*_*p, U*_. This result indicates that the peak behavior of *C*_*p*_ is primarily dominated by internal energy fluctuations. Since the kinetic contribution to the internal energy varies smoothly and monotonically with temperature, the anomalous maximum of *C*_*p*_ is governed by fluctuations in *U*_conf_. Specifically, the fluctuations in *U*_conf_ primarily originate from the topological breaking and local rearrangement of the hydrogen bond network.^22,41,45^ Such microscopic dynamics typically manifest as the local reorientation of water molecules and the distortion of adjacent hydrogen bonds. Notably, these topological rearrangements proceed via coordinated molecular jumps^46^ that persist even under high-pressure confinement.^47^ Crucially, such localized dynamics require only a small free volume, indicating a relatively weak dependence on macroscopic volume changes.^48^

**Figure 2:**
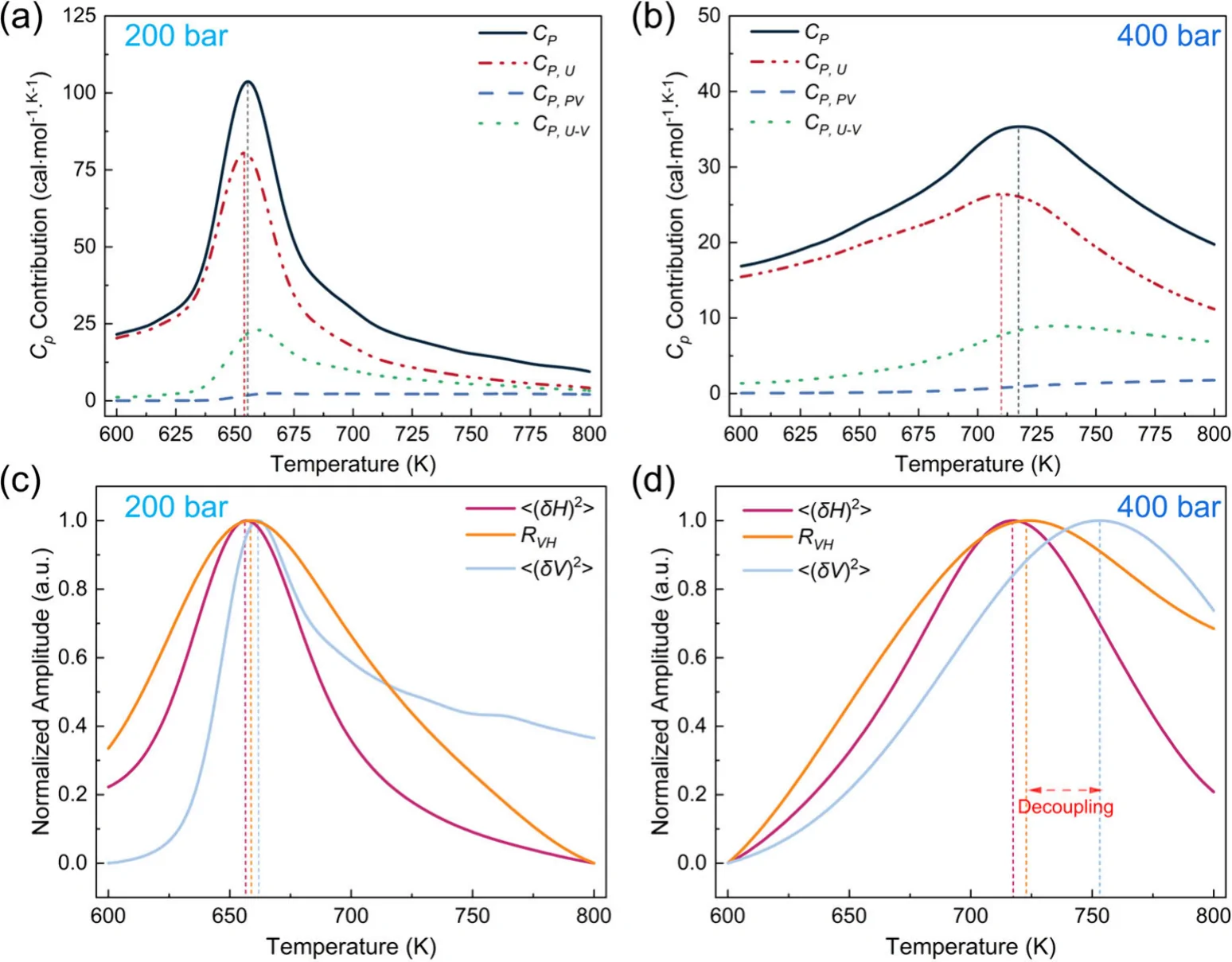
(a, b) Temperature dependence of *C*_*p*_ and its microscopic decomposition at 200 and 400 bar, including *C*_*p, U*_, *C*_*p, PV*_, and *C*_*p, U–V*_. (c, d) Normalized enthalpy fluctuations, volume fluctuations, and their cross-correlation coefficient *R*_*VH*_ at 200 and 400 bar. Vertical dashed lines mark response maxima. The curves were interpolated at 1 K intervals via cubic spline interpolation. For fluctuation data, an additional Savitzky-Golay filter was applied for clarity.

The value of α_*p*_ relates to the cross-fluctuations between volume and enthalpy ⟨*δHδV*⟩ of the system. To quantitatively evaluate the coupling degree between energy fluctuations and volume fluctuations, the cross-correlation coefficient *R*_*VH*_ (see SI Appendix Section S2) was further analyzed. As shown in Figure 2c and d, where all quantities are normalized to the [0, 1] range for direct comparison of their peak positions, the maximum of *R*_*VH*_ remains highly consistent with the peak of enthalpy fluctuations across both the near-critical low-pressure and high-pressure confined regions. This synchronous evolution underlies the persistent coincidence of the α_*p*_ and *C*_*p*_ maxima ridge lines throughout the supercritical region. From a microscopic perspective, the breaking and reforming of the hydrogen-bond network not only triggers significant changes in *U*_conf_, but also synergistically perturbs the local molecular arrangement, fundamentally driving the strong coupling between enthalpy and volume fluctuations. Notably, while the peak positions remain synchronized, the *R*_*VH*_ curve broadens considerably at higher pressures. This broadening indicates that the temperature window over which enthalpy and volume fluctuations remain appreciably correlated widens progressively far from the critical point, which is consistent with the transformation from a sharp near-critical crossover to a more gradual transition under high-pressure confinement.

In contrast, *K*_*T*_ relates to the volume self-fluctuations ⟨(*δV*)^2^⟩ of the system. As shown in Figure 2d, at 400 bar, the peak temperature of the volume fluctuations is significantly higher than that of the enthalpy fluctuations. This microscopic decoupling of fluctuation peaks reflects the distinct physical origins of the respective response maxima. Unlike the *C*_*p*_ maximum, which is driven by localized *U*_conf_ fluctuations and remains relatively insensitive to macroscopic volume changes, the peak of *K*_*T*_ arises directly from macroscopic volume fluctuations and is subject to different constraints. Microscopically, local volume contraction is resisted by a rapid increase in short-range intermolecular repulsion, while local volume expansion is limited by the external pressure work (*PδV*). Both constraints are significantly amplified under high pressure, so that the temperature must be further increased to overcome them and allow volume fluctuations to attain their maximum values, thereby shifting the *K*_*T*_ maximum to substantially higher temperatures than that of *C*_*p*_. When evaluating this macroscopic response, it is also important to distinguish the shift of the peak position from the amplitude behavior. At both 200 and 400 bar, the fluctuation amplitude decreases with increasing temperature as the system moves further from the critical region, which is a separate trend from the pressure-induced peak shift.

The above macroscopic response functions and statistical fluctuation behaviors have systematically revealed the thermodynamic state evolution of SCW. To quantitatively characterize solvent cohesive strength and cavity-formation thermodynamics in SCW, the cohesive energy density (CED) and the cavity-formation free energy, Δ*G*_cav_, are introduced as complementary descriptors. CED, defined in eq 5, quantifies the total energy density required to overcome all intermolecular interactions for complete molecular separation, which represents the overall cohesive strength of the fluid. Moreover, the internal pressure, *P*_int_, is also an important indicator of fluid cohesion.^49^ In the current work, *P*_int_ derived from the IAPWS-95 equation of state is used as an external thermodynamic reference for assessing the consistency of the cohesion-attenuation trend that is captured by the simulations (see SI Appendix Section S3). In contrast, Δ*G*_cav_(*r*), defined in eq 6, directly quantifies the free-energy penalty for creating a cavity of radius *r* and may be formally decomposed into internal-energy, entropy, and pressure–volume contributions.

5 $$CED=-\frac{E_{coh}}{V_{m}}$$

6 $$\Delta G_{cav}\left(r\right)=\Delta U_{cav}\left(r\right)-T\Delta S_{cav}\left(r\right)+P\Delta V_{cav}\left(r\right)$$

where *E*_coh_ is the cohesive energy, evaluated as – *U*_conf_ in the MD simulations, *V*_m_ is the molar volume, and Δ*U*_cav_, Δ*S*_cav_, and Δ*V*_cav_ are the internal energy change, entropy change, and volume change associated with cavity formation, respectively.

The above descriptors were derived from analyses of the MD trajectories. Since traditional macroscopic estimates of cohesive energy based on the latent heat of phase transitions or enthalpy of vaporization are not applicable in the supercritical region,^35,36,50^ CED was directly calculated from the MD-derived *U*_conf_. For each thermodynamic state, the positive available-radius distribution was obtained using random-probe analysis. Because the local free-volume environment changes markedly over the investigated *P-T* range, a cavity radius selected a priori and held constant across all thermodynamic states does not necessarily represent the same physical perturbation. Therefore, for each thermodynamic state, the mean available cavity radius was determined separately from the corresponding positive available-radius distribution and adopted as the characteristic cavity size for that state, as defined in eq 7:

7 $${\mathrm{R̅}}_{void}\left(T,P\right)=\underset{i}{\sum }\omega_{i}\left(T,P\right)r_{i}$$

where *r*_*i*_ is the center of the *i*-th positive-radius bin, and ω_*i*_(*T*,*P*) is the normalized probability weight of that bin in the positive available-radius distribution. The cavity-formation free energy evaluated at the state-dependent mean available cavity radius, denoted $\Delta G_{cav}^{\mathrm{R̅}}$ , was then calculated according to eq 8:

8 $$\Delta G_{cav}^{\mathrm{R̅}}\left(T,P\right)=-k_{B}T\;\ln P\left[R\geq {\mathrm{R̅}}_{void}\left(T,P\right)\right]$$

where *R* denotes the local available cavity radius at an individual random probe point. This approach directly relates the cavity size used in the free-energy calculation to the state-dependent local free-volume environment of water (see SI Appendix Section S4).

Figure 3a and b shows the isobaric evolution of CED and $G_{cav}^{\mathrm{R̅}}$ , respectively, over the investigated *P–T* range. With increasing pressure, the temperature-dependent behaviors of both quantities change markedly. Along the lower-pressure isobars, where the system approaches the critical region more closely, CED decreases rapidly upon heating, reflecting the rapid weakening of solvent-network cohesive strength. Meanwhile, $\Delta G_{cav}^{\mathrm{R̅}}$ also varies strongly with temperature, indicating a pronounced thermal sensitivity of the cavity-formation penalty near the critical region. As the isobaric paths shift to higher pressures and move farther away from the critical region, the variations in CED and $\Delta G_{cav}^{\mathrm{R̅}}$ become more gradual and extend over progressively broader temperature ranges. This broadening indicates that the thermodynamic responses associated with solvent cohesion and cavity formation evolve from sharp near-critical changes into continuous supercritical crossover behavior.

**Figure 3:**
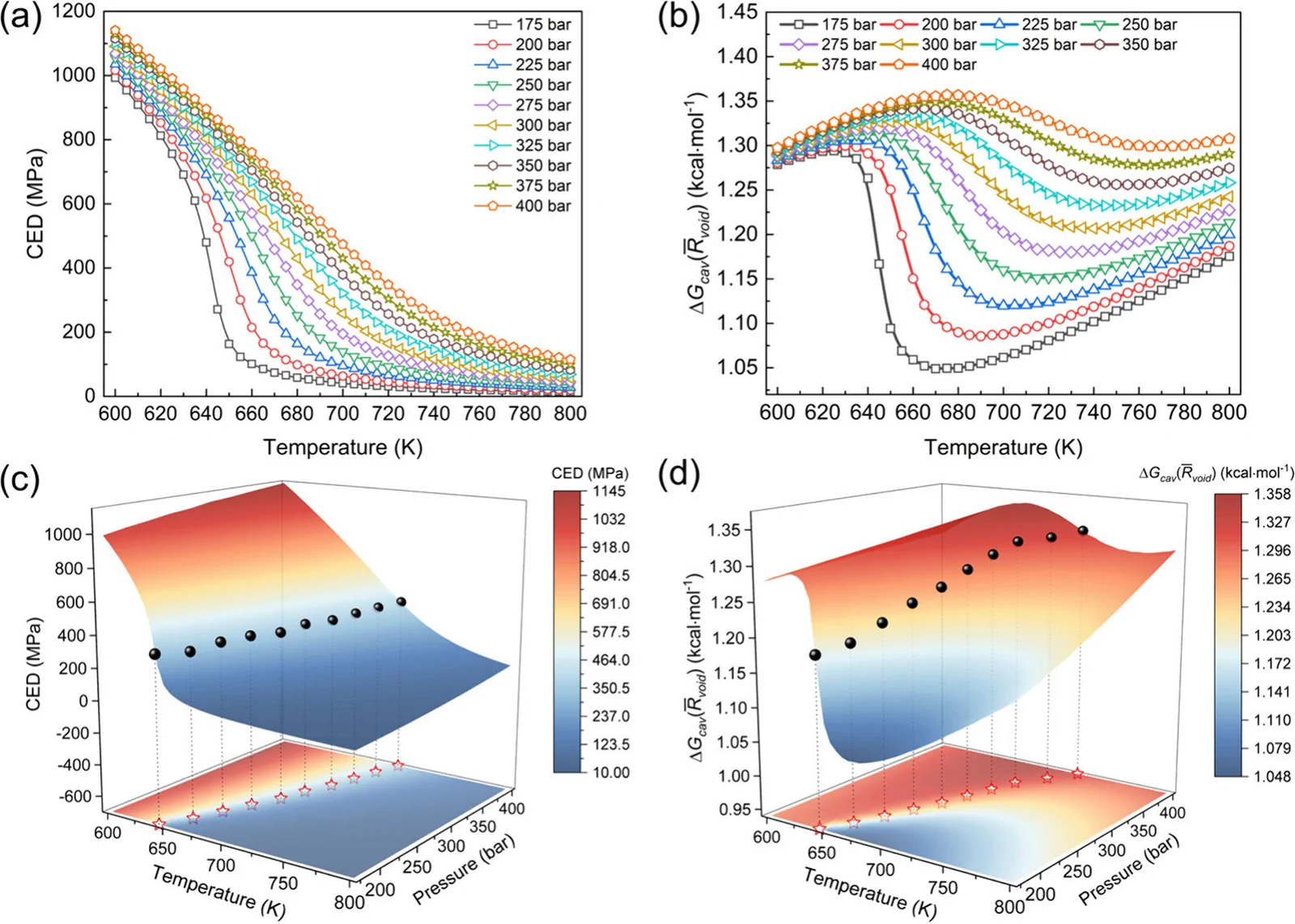
Isobaric evolution and *P–T* surfaces of solvent cohesion and the state-dependent mean-radius cavity-formation free energy, $\Delta G_{cav}^{\mathrm{R̅}}$ , in SCW. (a) Isobaric evolution curves of CED. (b) Isobaric evolution curves of $\Delta G_{cav}^{\mathrm{R̅}}$ . (c, d) *P–T* surfaces of CED and $\Delta G_{cav}^{\mathrm{R̅}}$ , respectively. The black circles in (c) and (d) denote the maxima of the corresponding thermal response coefficients, χ_CED_ and χ_cav_, respectively, and the red stars on the lower *P–T* planes represent their vertical projections.

To identify the characteristic temperatures at which CED and $\Delta G_{cav}^{\mathrm{R̅}}$ decrease most rapidly during isobaric heating, their corresponding thermal response coefficients, χ_CED_ and χ_cav_, were defined as shown in eqs 9 and 10, respectively. Their maxima trace the cohesive-response and cavity-response ridges, respectively, locating the thermodynamic states at which solvent-network cohesion and $\Delta G_{cav}^{\mathrm{R̅}}$ decrease most rapidly. Unlike traditional response functions, such as *C*_*p*_, α_*p*_, and *K*_*T*_, which reflect mixed contributions from different thermodynamic fluctuations, these coefficients specifically characterize the thermal responses of solvent cohesion and cavity-formation free energy. The thermodynamic interpretation of χ_CED_ based on the Maxwell relations^49^ and fluctuation theory^51^ is provided in SI Appendix Section S3.

9 $$\chi_{CED}=-{\left(\frac{\partial CED}{\partial T}\right)}_{P}$$

10 $$\chi_{cav}=-{\left(\frac{d\Delta G_{cav}^{\mathrm{R̅}}}{dT}\right)}_{P}$$

Figures 3c and d further illustrate the nonlinear evolution of CED and $\Delta G_{cav}^{\mathrm{R̅}}$ in *P–T* space. The χ_CED_ and χ_cav_ maxima are shown as black circles on the corresponding thermodynamic surfaces and are vertically projected onto the lower *P–T* planes as red stars, thereby visualizing the cohesive-response and cavity-response ridges. These two thermodynamic surfaces provide complementary visualizations of the supercritical crossover from the perspectives of bulk solvent cohesive strength and cavity formation. The state dependence of the characteristic cavity size is further demonstrated by the isobaric evolution of the mean available cavity radius and representative positive available-radius distributions shown in SI Appendix Section S4 and Figures S3 and S4.

Next, the maximum trajectories of χ_CED_ and χ_cav_ were projected onto the *P–T* plane and compared with the classical WL trajectories defined by the maxima of *C*_*p*_, α_*p*_ and *K*_*T*_ (Figure 4). Near the critical point, the cohesive-response and cavity-response trajectories remain closely aligned and converge toward the classical WL. The mutual alignment of the two response trajectories indicates that the rates of solvent-cohesion attenuation and reduction in $\Delta G_{cav}^{\mathrm{R̅}}$ reach their maxima at nearly the same thermodynamic states. Their convergence with the classical WL further shows that, in the near-critical region, the WL also marks the thermodynamic states associated with these responses, thereby broadening its physical interpretation and providing a new thermodynamic perspective on the liquid-like-to-gas-like crossover of SCW. At higher pressures far from the critical point, the two response trajectories become increasingly separated. The χ_CED_ maximum remains on the low-temperature side of the *C*_*p*_ maximum, whereas the χ_cav_ maximum shifts progressively toward higher temperatures. At 375 and 400 bar, the cavity-response maximum occurs at a higher temperature than the *C*_*p*_ maximum. These features reveal a distinct temperature ordering of thermodynamic responses during the high-pressure evolution of SCW.

**Figure 4:**
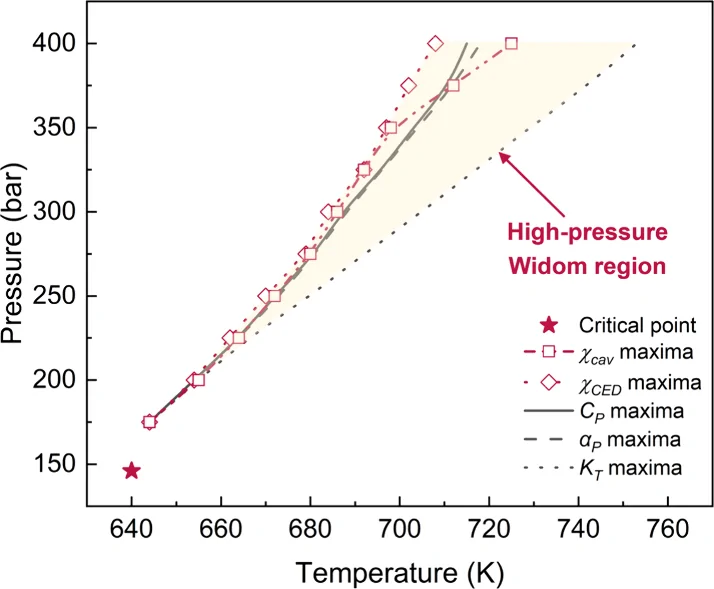
Relationship between the cohesive-response and cavity-response trajectories and the classical WL in the *P–T* plane. The red open diamonds and squares denote the χ_CED_ and χ_cav_ maxima obtained from TIP4*P*/2005 simulations, respectively, and the red star denotes the critical point. The classical WL is defined by the maxima of *C*_*p*_ (solid line), α_*p*_ (dashed line), and *K*_*T*_ (dotted line). The shaded area indicates the high-pressure Widom region bounded by the χ_CED_ and *K*_*T*_ maxima trajectories. The curves were interpolated at 1 K intervals and smoothed via cubic spline interpolation.

It is proposed that, over the pressure range in which the two response trajectories remain closely aligned, the corresponding response maxima occur at temperatures below those at which hydrogen-bond breaking and reformation, configurational rearrangements, and *U*_conf_ fluctuations become most pronounced. In the high-pressure supercritical regime, intermolecular cohesive interactions impose significant spatial constraints on configurational rearrangements. When the decay rate of cohesive strength reaches a peak, as indicated by the maximum of χ_CED_, these constraints are partially relieved, thereby providing a more favorable configurational environment for the subsequent *U*_conf_ fluctuations at higher temperatures.^24^ These fluctuations are dominated by frequent hydrogen-bond breaking and reformation, corresponding to the *C*_*p*_ maximum. At still higher pressures, the separation of the cavity- and cohesive-response maxima indicates that bulk cohesion attenuation and cavity-formation thermodynamics acquire distinct characteristic temperatures, revealing their thermodynamic differentiation within the high-pressure solvation environment.

The above phenomenon highlights that the traditional single WL is not sufficient to fully characterize the state evolution of SCW in the high-pressure region far from the critical point, necessitating the introduction of the “Widom region” concept. This concept is physically complementary to the “Widom delta” proposed by Yoon et al.^16,28,30,52^ and Ha et al.^29^ based on microscopic structures. The Widom delta focuses on the geometric connectivity of molecular spatial configurations, identifying the continuous mixing and percolation transitions of liquid-like and gas-like components via Voronoi tessellation or machine learning, thereby effectively characterizing the spatial inhomogeneity of the fluid. Previous studies^16,28–30,52^ have confirmed that the ridge lines of classical response function maxima (*C*_*p*_, α_*p*_, *K*_*T*_) are located entirely within the Widom delta. To provide a quantitative evaluation of this relationship, the χ_CED_ maximum temperatures were further compared with the percolation-threshold boundaries of the Widom delta across the overlapping pressure range (see SI Appendix Section S5).

By contrast, along the isobaric heating paths examined in the current study, the transition from a “liquid-like” state (strong cohesion, constrained dynamics) to a “gas-like” state (weak cohesion, free transport) manifests as a continuous process with a distinct sequence. The χ_CED_ maximum trajectory defines the low-temperature boundary of this region, marking the thermodynamic states at which solvent cohesion attenuates most rapidly. With further heating into the interior of the region, *C*_*p*_ and α_*p*_ (dominated by *U*_conf_ fluctuations and structural rearrangements) reach their maxima synergistically. The χ_*cav*_ maximum trajectory, which marks the thermodynamic states at which $\Delta G_{cav}^{\mathrm{R̅}}$ decreases most rapidly, also lies within this region: it remains close to the χ_CED_ maximum trajectory over the lower-to-intermediate pressure range but shifts progressively toward higher temperatures with increasing pressure. Finally, *K*_*T*_ (dominated by volume fluctuations) peaks at even higher temperatures, bounding the high-temperature side. This finding clarifies the thermodynamic response sequence across the high-pressure “Widom region” from the perspectives of solvent cohesion and cavity formation, and provides a thermodynamic basis for understanding the solvation properties of SCW.

In conclusion, the current study first examined the thermodynamic responses of SCW along isobars and clarified the origin of the high-pressure divergence of the classical WL from the perspective of statistical fluctuations and intermolecular interactions. It is demonstrated that the maxima of *C*_*p*_ and α_*p*_ remain highly synchronized, a phenomenon originating primarily from *U*_conf_ fluctuations and structural rearrangements driven by the breaking and reformation of the hydrogen-bond network. In contrast, the maximum of *K*_*T*_ shifts to significantly higher temperatures. This deviation occurs because high-pressure conditions suppress volume fluctuations through amplified short-range repulsion and external pressure work, shifting the maximum volume fluctuation to substantially higher temperatures than the *C*_*p*_ and α_*p*_ maxima, which are dominated by *U*_conf_ fluctuations.

Subsequently, the thermodynamic evolution of solvent cohesive strength and cavity formation in SCW was examined along isobars. The newly introduced χ_CED_ and χ_cav_ response trajectories identify the thermodynamic states at which solvent-network cohesive strength attenuates most rapidly and the cavity-formation free energy decreases most rapidly, respectively. The maximum trajectories of χ_CED_ and χ_cav_ closely coincide with each other and with the classical WL near the LGCP but become progressively separated at high pressures. On this basis, a high-pressure Widom region is defined, with the χ_CED_ maximum trajectory forming its low-temperature boundary, the *C*_*p*_, α_*p*_ and χ_cav_ maximum trajectories evolving within the region, and the *K*_*T*_ maximum trajectory forming its high-temperature boundary. A comparison with the structural Widom delta further indicates that this thermodynamic description is broadly consistent with previous structural interpretations. Ultimately, the thermodynamic framework established herein offers a perspective on solvent cohesion and cavity formation for understanding the solvation behavior of SCW. Beyond advancing the fundamental understanding of phase-like crossover behavior in supercritical fluids, this framework provides a thermodynamic basis for screening *P-T* operating windows that may favor solute accommodation and transport in SCW-based separation and reaction processes.

## Computational Methods

All MD simulations were performed using the TIP4P/2005 force field,^42^ which has been extensively validated for SCW thermodynamics and WL behavior.^23^ NIST benchmark data^43^ based on the IAPWS-95 equation of state^53^ were used as an external reference for the classical WL behavior, and the EoS-derived *P*_int_ analysis was included as an additional thermodynamic reference. Details of the MD simulations, fluctuation analyses, response derivations, cavity-formation free energy calculations, and the comparison with the structural Widom delta are provided in the Supporting Information (Sections S1–S5).

## Supplementary Material



## Notes

Detailed MD simulation parameters, NIST benchmark data extraction methods, fluctuation and response-coefficient analyses, cavity-formation free energy calculations, and the comparison with the structural Widom delta (PDF)

## References

[1] Weingärtner H., Franck E. U. (2005). Supercritical Water as a Solvent. Angew. Chem., Int. Ed..

[2] Peng B., Zhang K., Sun Y., Han B., He M. (2025). Role of Water
in Green Carbon Science. J. Am. Chem. Soc..

[3] Qian L., Wang S., Xu D., Guo Y., Tang X., Wang L. (2016). Treatment of Municipal
Sewage Sludge in Supercritical Water: A Review. Water Res..

[4] Peplow M. (2025). Can This Revolutionary
Plastics-Recycling Plant Help Solve the Pollution Crisis?. Nature.

[5] Peterson A. A., Vogel F., Lachance R. P., Fröling M., Antal M. J., Tester J. W. (2008). Thermochemical Biofuel
Production in Hydrothermal Media: A Review of Sub- and Supercritical
Water Technologies. Energy Environ. Sci..

[6] Okolie J. A., Nanda S., Dalai A. K., Berruti F., Kozinski J. A. (2020). A Review
on Subcritical and Supercritical Water Gasification of Biogenic, Polymeric
and Petroleum Wastes to Hydrogen-Rich Synthesis Gas. Renewable Sustainable Energy Rev..

[7] Adschiri T., Lee Y.-W., Goto M., Takami S. (2011). Green Materials
Synthesis
with Supercritical Water. Green Chem..

[8] Chan H.-K., Kwok P. C. L. (2011). Production Methods
for Nanodrug Particles Using the
Bottom-up Approach. Adv. Drug Delivery Rev..

[9] Scott S., Driesner T., Weis P. (2015). Geologic Controls on Supercritical
Geothermal Resources above Magmatic Intrusions. Nat. Commun..

[10] Hu Y., Gong M., Xing X., Wang H., Zeng Y., Xu C. C. (2020). Supercritical Water
Gasification of Biomass Model Compounds: A Review. Renewable Sustainable Energy Rev..

[11] Zhao Q., Niu J., Dong Y., Song Z., Ke B., An P., Jin H., Guo L. (2024). Sub- and Supercritical
Water Upgrading of Heavy Oil:
A Review of Laboratory-Scale Research on Upgrading Performance and
Physicochemical Mechanism. Chem. Eng. J..

[12] Niu J., Zhao Q., Zheng L., Ke B., Song Z., Chen Y., Jin H., Adams M. J., Guo L. (2026). Predicting
the Solvent Properties of Supercritical Water by Integrating Solubility
Parameter Theory and Molecular Force Fields. Adv. Chem. Eng..

[13] Atkins, P. ; de Paula, J. ; Keeler, J. Atkins’ Physical Chemistry, 11th ed.; Oxford University Press: Oxford, U.K., 2018.

[14] Cockrell C., Brazhkin V. V., Trachenko K. (2021). Transition
in the Supercritical State
of Matter: Review of Experimental Evidence. Phys. Rep..

[15] Li X., Jin Y. (2024). Thermodynamic
Crossovers in Supercritical Fluids. Proc. Natl.
Acad. Sci. U.S.A..

[16] Ha M. Y., Yoon T. J., Tlusty T., Jho Y., Lee W. B. (2020). Universality
Scaling, and Collapse in Supercritical Fluids. J. Phys. Chem. Lett..

[17] Luo J., Xu L., Lascaris E., Stanley H. E., Buldyrev S. V. (2014). Behavior of the
Widom Line in Critical Phenomena. Phys. Rev.
Lett..

[18] Widom B. (1965). Surface Tension
and Molecular Correlations near the Critical Point. J. Chem. Phys..

[19] Widom B. (1965). Equation of
State in the Neighborhood of the Critical Point. J. Chem. Phys..

[20] McMillan P. F., Stanley H. E. (2010). Going Supercritical. Nat. Phys..

[21] Simeoni G. G., Bryk T., Gorelli F. A., Krisch M., Ruocco G., Santoro M., Scopigno T. (2010). The Widom Line as the Crossover between
Liquid-like and Gas-like Behaviour in Supercritical Fluids. Nat. Phys..

[22] Gallo P., Corradini D., Rovere M. (2014). Widom Line and Dynamical Crossovers
as Routes to Understand Supercritical Water. Nat. Commun..

[23] Corradini D., Rovere M., Gallo P. (2015). The Widom Line and Dynamical Crossover
in Supercritical Water: Popular Water Models versus Experiments. J. Chem. Phys..

[24] Maxim F., Karalis K., Boillat P., Banuti D. T., Damian J. I. M., Niceno B., Ludwig C. (2021). Thermodynamics and Dynamics of Supercritical
Water Pseudo-Boiling. Adv. Sci..

[25] Maxim F., Contescu C., Boillat P., Niceno B., Karalis K., Testino A., Ludwig C. (2019). Visualization
of Supercritical Water
Pseudo-Boiling at Widom Line Crossover. Nat.
Commun..

[26] Belyakov M. Yu., Kulikov V. D. (2025). Asymptotic Widom Line of Supercritical Fluid within
the Scaling Theory. J. Supercrit. Fluids.

[27] Shagurin A., Miannay F. A., Kiselev M. G., Jedlovszky P., Affouard F., Idrissi A. (2024). Widom Line in Supercritical
Water
in Terms of Changes in Local Structure: Theoretical Perspective. J. Phys. Chem. Lett..

[28] Yoon T. J., Ha M. Y., Lee W. B., Lee Y.-W. (2018). Probabilistic Characterization
of the Widom Delta in Supercritical Region. J. Chem. Phys..

[29] Ha M. Y., Yoon T. J., Tlusty T., Jho Y., Lee W. B. (2018). Widom Delta
of Supercritical Gas–Liquid Coexistence. J. Phys. Chem. Lett..

[30] Yoon T. J., Ha M. Y., Lee W. B., Lee Y.-W. (2019). A Corresponding-State
Framework for the Structural Transition of Supercritical Fluids across
the Widom Delta. J. Chem. Phys..

[32] Ben-Naim, A. Solvation Thermodynamics; Springer US: Boston, MA, 1987.

[33] Marcus Y. (2013). Internal Pressure
of Liquids and Solutions. Chem. Rev..

[34] Hildebrand J. H., Wood S. E. (1933). The Derivation
of Equations for Regular Solutions. J. Chem.
Phys..

[35] Hansen C. M. (2004). 50 Years
with Solubility Parameters—Past and Future. Prog. Org. Coat..

[36] Hansen, C. M. Hansen Solubility Parameters: A User’s Handbook, 2nd ed.; CRC Press: Boca Raton, 2007.

[37] Dack M. R. J. (1975). The
Importance of Solvent Internal Pressure and Cohesion to Solution Phenomena. Chem. Soc. Rev..

[38] Marcus Y. (2020). The Structure
of Mixtures of Water and Acetone Derived from Their Cohesive Energy
Densities and Internal Pressures. J. Mol. Liq..

[39] Otto S. (2013). The Role of
Solvent Cohesion in Nonpolar Solvation. Chem.
Sci..

[40] Acree W. E., Panayiotou C. (2026). On Dispersion and Polar Interactions and Solvation
and Cohesion Energies. A First Round. Fluid
Phase Equilib..

[41] Gallo P., Amann-Winkel K., Angell C. A., Anisimov M. A., Caupin F., Chakravarty C., Lascaris E., Loerting T., Panagiotopoulos A. Z., Russo J., Sellberg J. A., Stanley H. E., Tanaka H., Vega C., Xu L., Pettersson L. G. M. (2016). Water:
A Tale of Two Liquids. Chem. Rev..

[42] Abascal J. L. F., Vega C. (2005). A General Purpose Model for the Condensed Phases of
Water: TIP4P/2005. J. Chem. Phys..

[43] Huber M. L., Lemmon E. W., Bell I. H., McLinden M. O. (2022). The NIST REFPROP
Database for Highly Accurate Properties of Industrially Important
Fluids. Ind. Eng. Chem. Res..

[44] Allen, M. P. ; Tildesley, D. J. Computer Simulation of Liquids, 2nd ed.; Oxford University Press: Oxford, 2017.

[45] Skarmoutsos I., Henao A., Guardia E., Samios J. (2021). On the Different Faces
of the Supercritical Phase of Water at a Near-Critical Temperature:
Pressure-Induced Structural Transitions Ranging from a Gaslike Fluid
to a Plastic Crystal Polymorph. J. Phys. Chem.
B.

[46] Laage D., Hynes J. T. (2006). A Molecular Jump Mechanism of Water
Reorientation. Science.

[47] Sahle C. J., Sternemann C., Schmidt C., Lehtola S., Jahn S., Simonelli L., Huotari S., Hakala M., Pylkkänen T., Nyrow A., Mende K., Tolan M., Hämäläinen K., Wilke M. (2013). Microscopic Structure of Water at Elevated Pressures and Temperatures. Proc. Natl. Acad. Sci. U.S.A..

[48] Choudhary A., Kundu A., Singh C., Sharma A., Pant K. K., Chandra A. (2025). Supercritical Water:
Density-Independent Angular Jumps. J. Phys.
Chem. B.

[49] Callen, H. B. Thermodynamics and an Introduction to Thermostatistics, 2nd ed.; John Wiley & Sons: New York, 1985.

[50] Marcus, Y. Supercritical Water: A Green Solvent, Properties and Uses; Wiley: Hoboken, NJ, 2012.

[51] Hockney, R. W. ; Eastwood, J. W. Computer Simulation Using Particles; CRC Press: Boca Raton, 2021.

[52] Yoon T. J., Ha M. Y., Lee W. B., Lee Y.-W. (2017). Monte Carlo
Simulations
on the Local Density Inhomogeneities of Sub- and Supercritical Carbon
Dioxide: Statistical Analysis Based on the Voronoi Tessellation. J. Supercrit. Fluids.

[53] Wagner W., Pruß A. (2002). The IAPWS Formulation 1995 for the
Thermodynamic Properties
of Ordinary Water Substance for General and Scientific Use. J. Phys. Chem. Ref. Data.

