# Supplementary material for: Crossing the Widom Line: Evolution of Cohesive Strength and Cavity Formation in Supercritical Water
Source: J Phys Chem Lett. 2026 Aug 3;17(32):9128–35. doi: 10.1021/acs.jpclett.6c01190 (PMC13488442; doi:10.1021/acs.jpclett.6c01190)
Supplement: Supplementary file 1 [file jz6c01190_si_001.pdf]

Supporting Information

Crossing the Widom Line: Evolution of Cohesive Strength and Cavity Formation in Supercritical Water

Jiacheng Niu [a], Qiuyang Zhao [a, *], Michael J. Adams [b, *], Hao Lu [a], Yin Chen [a], Xinjian Wang [a], Hui Jin [a] & Liejin Guo [a, *]

[a] *State Key Laboratory of Multiphase Flow in Power Engineering, Xi'an Jiaotong University, Xi'an, Shaanxi 710049, People's Republic of China*
[b] *School of Chemical Engineering, University of Birmingham, Birmingham B15 2TT, United Kingdom*

[*]Corresponding author.
E-mail addresses: qyzhao@mail.xjtu.edu.cn (Q. Zhao), m.j.adams@bham.ac.uk (M. Adams), lj-guo@mail.xjtu.edu.cn (L. Guo).

Contents

- Section S1: Computational methods and NIST benchmark data extraction.
- Section S2: Calculation of microscopic fluctuations and cross-correlations.
- Section S3: Thermodynamic details of internal pressure, cohesive energy density, and thermal response coefficients.
- Section S4: Random-probe analysis and calculation of the state-dependent mean-radius cavity-formation free energy.
- Section S5: Approximate quantitative comparison between the χ_{CED} response ridge and the Widom-delta boundaries.

Section S1: Computational methods and NIST benchmark data extraction.

MD simulations were performed in LAMMPS[1] using the TIP4P/2005 force field,[2] which reliably reproduces the critical behavior of water (T_c = 640 K, P_c = 146 bar, ρ_c = 310 kg/m^3). [3,4] This model was selected because prior comparative studies have shown that it provides the best overall agreement with experimental Widom-line trajectories and thermodynamic response functions among several common water models.[5] While advanced polarizable or many-body potentials (e.g., MB-pol) offer enhanced accuracy for specific hydrogen-bond properties, their computational cost is prohibitive for the extensive thermodynamic grid mapping required in the current work, which entails an aggregate of approximately 800 ns of trajectory sampling across the (P, T) phase diagram. Regarding the potential sensitivity to molecular flexibility, the TIP4P/2005 model employs a rigid water geometry. Although it omits high-frequency intramolecular vibrational modes, prior benchmarking has demonstrated that this rigid framework faithfully reproduces the synchronous C_p, α_p maxima and the delayed K_T maximum observed in experiment.[5] This supports the adequacy of a rigid geometry for capturing the intermolecular structural crossover that dominates the supercritical Widom region. Therefore, the qualitative trends discussed in this work are physically meaningful and representative of SCW.

All simulations were implemented in the NPT ensemble with Nosé-Hoover thermostats/barostats, a 1 fs time step, and 3D periodic boundary conditions for a 2048-water-molecule system. Data were collected along 175–400 bar isobars (ΔP = 25 bar) over 600–800 K (ΔT = 5 K), with each state point simulated for 2000 ps (100 ps equilibration, 1900 ps sampling). A 10 Å cutoff was applied for short-range interactions, long-range electrostatics were calculated by employing the PPPM algorithm,[6] and the rigid water structure was constrained with the SHAKE algorithm.[7]

NIST benchmark data were obtained from the REFPROP software,[8] which is based on the high-precision IAPWS-95 equation of state (EoS).[9] Isobaric thermodynamic properties and P_{int} data were extracted over T = 600-800K (ΔT = 5 K) and P = 245-470 bar (ΔP = 25 bar). The critical parameters for pure water are T_c = 647.10 K, P_c = 220.64 bar, and ρ_c = 322 kg/m^3.

For the thermodynamic state functions (enthalpy, density, cohesive energy, internal pressure), the raw data were interpolated at 1 K intervals using cubic spline interpolation to generate smooth, continuous curves. For the fluctuation data (enthalpy fluctuations, volume fluctuations, cross-correlation coefficients), a Savitzky–Golay filter was applied after cubic spline interpolation to improve the signal-to-noise ratio. The characteristic peak positions were resolved to within approximately half the 5 K sampling interval by spline interpolation. Therefore, the upper bound on the temperature uncertainty arising from the finite sampling resolution is approximately ±2.5 K. This estimate reflects the maximum discretization error and does not incorporate additional systematic uncertainties inherent to the force field or the EoS formulation.

Section S2: Calculation of microscopic fluctuations and cross-correlations.

To systematically evaluate the volume self-fluctuations, enthalpy self-fluctuations, and the cross-correlation behavior under varying pressures, the relevant thermodynamic data were extracted directly from the MD trajectories generated in the NPT ensemble. The specific statistical and computational methods are as follows:

a) Statistical variance calculation:

According to the fluctuation-dissipation theorem, the variances of volume and enthalpy are directly related to the isothermal compressibility and the isobaric heat capacity, respectively. These fluctuation variances were calculated using Equations S1 and S2:

$$\langle(\delta V)^2\rangle = \langle V^2\rangle - \langle V\rangle^2 \tag{S1}$$

$$\langle(\delta H)^2\rangle = \langle H^2\rangle - \langle H\rangle^2 \tag{S2}$$

where ⟨...⟩ denotes the ensemble average over the production stage of the simulation at a given temperature.

b) Cross-correlation coefficient:

The degree of coupling between volume fluctuations and enthalpy fluctuations was quantified by the Pearson correlation coefficient (R_{VH}), defined as:

$$R_{VH} = \frac{\langle \delta V \delta H\rangle}{\sqrt{\langle(\delta V)^2\rangle\langle(\delta H)^2\rangle}} = \frac{\langle VH\rangle - \langle V\rangle\langle H\rangle}{\sigma_V \sigma_H} \tag{S3}$$

where σ_V and σ_H represent the standard deviations of volume and enthalpy, respectively.

c) Data normalization:

To intuitively compare the peak positions and evolutionary profiles of the different physical quantities, all relevant fluctuation metrics were scaled to the [0, 1] range using the min-max normalization method:

$$Y_{norm} = \frac{Y - Y_{\min}}{Y_{\max} - Y_{\min}} \tag{S4}$$

where Y represents a specific physical quantity at a given temperature, while Y_{min} and Y_{max} denote the minimum and maximum values of that quantity over the entire investigated temperature range, respectively.

Notably, this linear scaling preserves the relative functional profiles and, more critically, the peak locations of the raw data. Consequently, all conclusions drawn from the comparison of peak

positions remain unchanged regardless of whether normalized or unnormalized amplitudes are used. This transformation acts strictly as a visual aid, enabling a direct comparison of the temperature-dependent curves within a single plot despite their distinct physical dimensions and vastly different absolute magnitudes.

Section S3: Thermodynamic details of internal pressure, cohesive energy density, and thermal response coefficients.

To supplement the main-text discussion of cohesive-strength evolution, this section distinguishes the thermodynamic interpretations of P_{int} and CED and derives their thermal response coefficients. P_{int} and CED both have dimensions of pressure and characterize intermolecular cohesion, but they describe different aspects of the cohesive behavior of the fluid. In the present work, the P_{int} values derived from the IAPWS-95 equation of state are used as an external thermodynamic reference for assessing the consistency of the cohesion-attenuation trend captured by the TIP4P/2005 simulations, whereas CED is calculated directly from the MD configurational potential energy and is used as the primary microscopic descriptor of solvent-network cohesion.

In classical thermodynamics, internal pressure is defined as the isothermal partial derivative of internal energy with respect to volume:

$$P_{\text{int}} = \left(\frac{\partial U}{\partial V} \right)_T \tag{S5}$$

Under isothermal conditions, the average kinetic energy is fixed by equipartition for a given number of degrees of freedom; therefore, the volume dependence of the internal energy is mainly associated with changes in the intermolecular configurational contribution, U_{conf}. Such a relationship enables P_{int} to sensitively reflect the variation of U_{conf} under infinitesimal volume perturbations. [10] In contrast, CED quantifies the total energy density required to overcome all intermolecular interactions for complete molecular separation, which represents the overall cohesive strength of the fluid. Thus, both P_{int} and CED can be regarded as pressure-dimensional cohesion descriptors, although they describe different aspects of fluid cohesion.

Distinguishing between these two cohesion descriptors is particularly important for hydrogen-bonded fluids. Highly directional hydrogen-bond networks possess significant spatial topological flexibility. When subjected to small volume perturbations or subtle rearrangements, the system preferentially buffers energy changes through bond angle relaxation or local orientational rearrangements.[11] Such flexibility can partially dampen the response of P_{int} to changes within the hydrogen-bond network. Therefore, the evolutionary characteristics of P_{int} are expected to be more sensitive to non-directional interactions. These include repulsive and dispersion interactions, which lack the structural flexibility of hydrogen bonding and remain highly sensitive to volume changes.

In contrast, CED comprehensively characterizes the contributions of all intermolecular interactions within the system. In particular, it captures the dominant contribution of the hydrogen-bond network.[12–14]

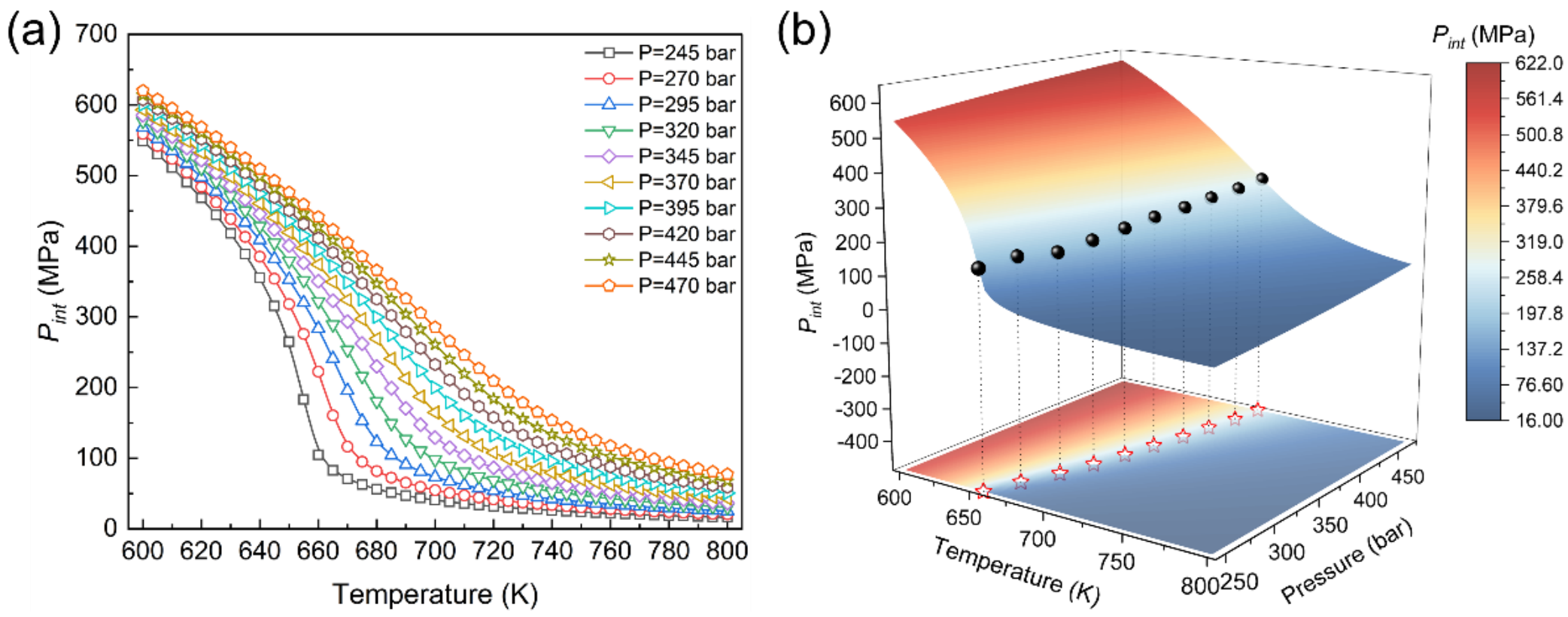

Figure S1. Internal-pressure analysis of SCW based on the IAPWS-95 equation of state. (a) Isobaric evolution curves of P_{int} for SCW. (b) P–T–P_{int} surface within the supercritical region. The black circles on the surface denote the extrema of χ_{int} along each isobar, and the red stars on the lower P–T plane represent their vertical projections.

As shown in Figure S1a, P_{int} decreases monotonically along the isobaric heating paths. Near the critical region, the P_{int} curves exhibit a steep temperature-dependent decline, indicating that the internal-pressure contribution to fluid cohesion is highly sensitive to thermal perturbations. With increasing pressure, this steep variation gradually evolves into a smoother and more continuous attenuation behavior, which is consistent with the progressive broadening of the near-critical anomaly under high-pressure conditions. The P–T surface in Figure S1b further confirms the continuous decrease of P_{int} from the low to the high-temperature region across the investigated pressure range. The black circles mark the extrema of the thermal response coefficient χ_{int}, and the red stars represent their vertical projections onto the lower P-T plane. These extrema identify the characteristic states where the internal-pressure contribution to cohesion attenuates most rapidly.

According to the cyclic relation of partial derivatives, the isochoric thermal pressure coefficient can be transformed into the ratio of α_p to K_T:

$$\left(\frac{\partial P}{\partial T}\right)_V = -\frac{(\partial V / \partial T)_P}{(\partial V / \partial P)_T} = \frac{\alpha_P}{K_T} \tag{S6}$$

Substituting into the expression for P_{int} yields the classical relationship between internal pressure and conventional macroscopic response functions:

$$P_{\text{int}} = T\frac{\alpha_p}{K_T} - P \tag{S7}$$

The thermal response coefficient of the internal pressure, χ_{int}, is defined as the negative isobaric derivative of P_{int} with respect to temperature. Taking the total differential of this term under isobaric conditions, where $(\partial P/\partial T)_P = 0$, gives:

$$\chi_{\text{int}} = -\left(\frac{\partial P_{\text{int}}}{\partial T}\right)_P = -\left[\frac{\partial}{\partial T}\left(T\frac{\alpha_p}{K_T} - P\right)\right]_P \tag{S8}$$

Applying the product and quotient rules to expand the above equation yields:

$$\chi_{\text{int}} = -\left[\frac{\alpha_p}{K_T} + T\left(\frac{\partial(\alpha_p / K_T)}{\partial T}\right)_P\right] = -\frac{\alpha_p}{K_T} - \frac{T}{K_T}\left(\frac{\partial \alpha_p}{\partial T}\right)_P + \frac{T\alpha_p}{K_T^{\,2}}\left(\frac{\partial K_T}{\partial T}\right)_P \tag{S9}$$

This expression indicates that χ_{int} contains the temperature derivatives of α_p and K_T. Therefore, χ_{int} can be regarded as a higher-order thermodynamic derivative that tracks the temperature-dependent attenuation of the internal-pressure contribution to cohesion.

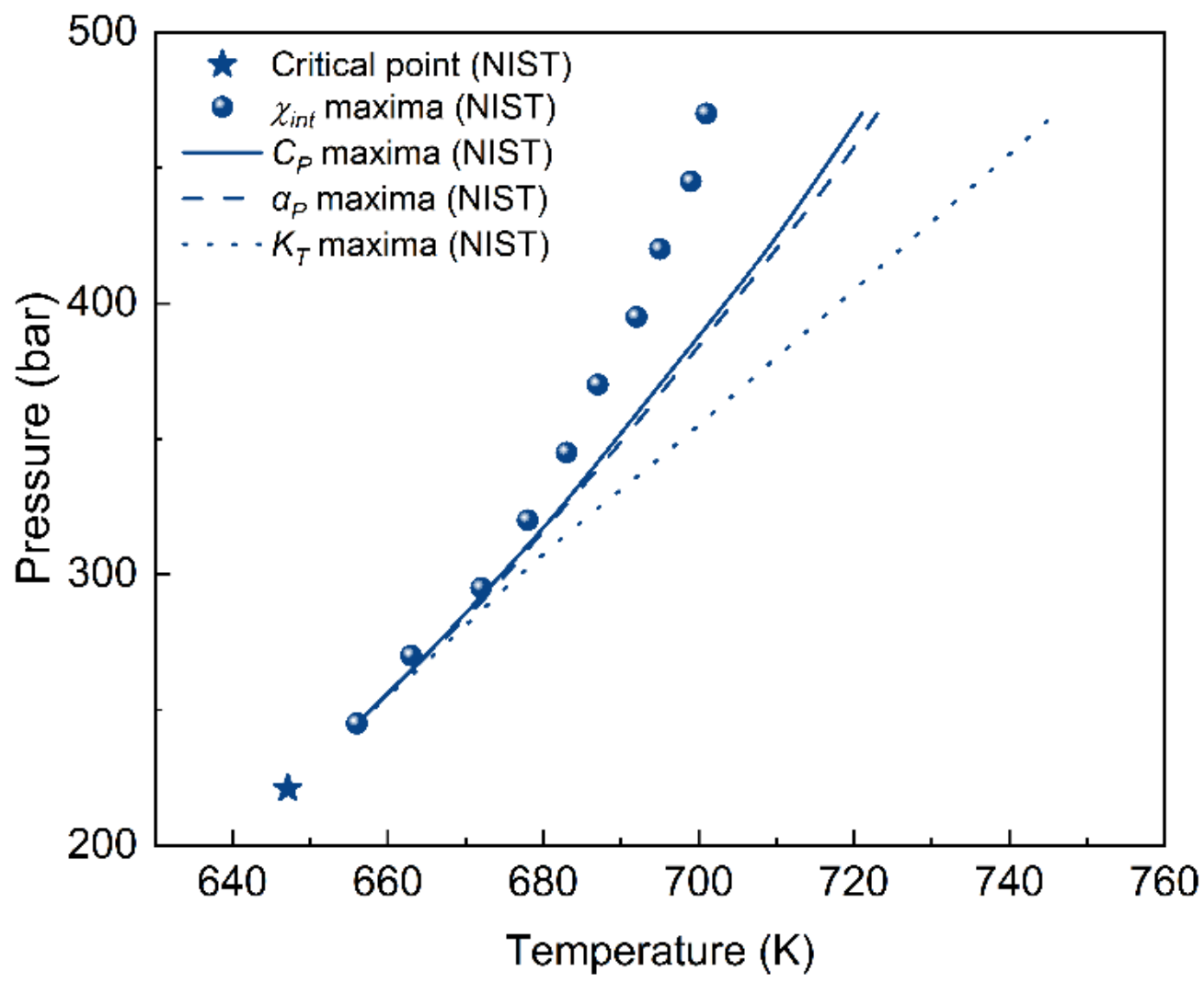

Figure S2. Comparison of the χ_{int} maximum trajectory and the classical WL trajectories in the *P*–*T* plane. Blue circles denote the χ_{int} maxima obtained from the IAPWS-95 equation of state. These points are plotted together with the critical point and the classical WL trajectories defined by the maxima of C_p (solid lines), α_p (dashed lines), and K_T (dotted lines). The curves were interpolated at 1 K intervals and smoothed via cubic spline interpolation.

Figure S2 shows the maximum trajectory of χ_{int} projected and compared with the classical WL defined by the maxima of C_p, α_p, and K_T. Upon approaching the critical point, the χ_{int} maximum trajectory naturally converges with the classical WL and remains closely aligned over a near-critical interval of approximately 30 K and 80 bar in the IAPWS-95 EoS framework. This convergence is

consistent with the universal scaling behavior near the critical point and indicates that, within this region, the classical WL also captures the thermodynamic states where the internal-pressure contribution to cohesion attenuates most rapidly. However. at higher pressures far from the critical point, the χ_{int} maximum trajectory diverges from the classical WL and shifts toward lower temperatures than the C_p maximum trajectory. This divergence indicates that the classical response-function maxima no longer coincide with the states of most rapid internal-pressure attenuation. Instead, the weakening of intermolecular cohesion reflected by χ_{int} occurs at lower temperatures, before the configurational and volumetric fluctuation maxima identified by the classical response functions are reached. This trend is consistent with the sequential-crossover representation discussed in the main text and provides an EoS-based thermodynamic reference for the simulation-derived χ_{CED} response ridge.

For the simulation-derived cohesion descriptor, CED is defined as the total energy density required to overcome all intermolecular interactions within the system for complete molecular separation. Within the framework of molecular dynamics simulations, this is equivalent to the negative intermolecular configurational potential energy, U_{conf}, per molar volume, V_m:

$$\mathrm{CED} = -\frac{U_{\mathrm{conf}}}{V_m} \tag{S10}$$

The CED thermal response coefficient, χ_{CED}, which is defined in the current study, is the negative isobaric partial derivative of CED with respect to temperature:

$$\chi_{CED} = -\left(\frac{\partial CED}{\partial T}\right)_P = \left[\frac{\partial}{\partial T}\left(\frac{U_{conf}}{V_m}\right)\right]_P \tag{S11}$$

Applying the quotient rule for differentiation, this can be expanded into two terms:

$$\chi_{CED} = \frac{1}{V_m}\left(\frac{\partial U_{conf}}{\partial T}\right)_P - \frac{U_{conf}}{V_m^{2}}\left(\frac{\partial V_m}{\partial T}\right)_P \tag{S12}$$

Two key physical contributions can be identified on the right-hand side of Eq. S12. The term $(\partial U_{conf}/\partial T)_P$ is associated with the configurational contribution to the isobaric heat capacity. In the microscopic fluctuation decomposition discussed in the main text, this is exactly the core driving term responsible for the intense fluctuations of the configurational potential energy (corresponding to the configurational portion of the internal energy self-fluctuation term $C_{p,U}$ in Figures 2a and b of the main manuscript). The factor $1/V_m(\partial V_m/\partial T)_P$ in the second term is precisely the macroscopic thermal expansion coefficient αp, while the remaining factor $-U_{conf}/V_m$ corresponds to the definition

of CED. Therefore, after simplification, χ_{CED} can be expressed in the following coupled form:

$$\chi_{CED} = \frac{1}{V_m} C_{p,conf} + CED \cdot \alpha_p \tag{S13}$$

The above analysis demonstrates that χ_{int} contains temperature-gradient terms related to thermal expansion and compressibility and can therefore be classified as a higher-order thermodynamic derivative. Furthermore, χ_{CED} can be rigorously decomposed into the coupling of a configurational heat capacity term and a volume expansion term. These derivations provide a thermodynamic basis for using χ_{int} and χ_{CED} as characteristic descriptors of cohesion attenuation.

Section S4: Random-probe analysis and calculation of the state-dependent mean-radius cavity-formation free energy.

To characterize the local void space in SCW, a random-probe analysis was performed using a custom Python script. For each thermodynamic state, 50,000 virtual probe points were randomly generated in each sampled configuration, with configurations extracted every 1 ps from the 1900 ps production trajectory. The distance from each probe point to the nearest water oxygen atom, $d_{nearest\_O}$, was determined using a nearest-neighbor search accelerated by a k-d tree data structure. The local available cavity radius, R, was defined as:

$$R = d_{nearest\_O} - r_{vdw} \tag{S14}$$

where r_{vdW} = 1.40 Å is the van der Waals radius of the oxygen atom.[15] A positive value of R represents the local radius available outside the oxygen excluded volume, whereas $R \leq 0$ indicates that the probe point lies within the excluded volume of a water molecule.

For a prescribed cavity radius r, the probability of finding a local available radius equal to or larger than r, denoted as $P(R \geqslant r)$, was calculated as the fraction of all random probe points satisfying this condition. The cavity-formation free energy at radius r was then evaluated using the standard statistical-mechanical relation:

$$\Delta G_{cav}(r;T,P) = -k_B T \ln P(R \geq r) \tag{S15}$$

where k_B is the Boltzmann constant and T is the absolute temperature.

Because the local free-volume environment changes markedly over the investigated P–T range, a cavity radius selected a priori and held constant across all thermodynamic states does not necessarily represent the same physical perturbation. Therefore, the mean available cavity radius was determined separately for each thermodynamic state from the corresponding positive available-radius distribution. The positive values of R were discretized into radius bins with centers r_i. If $q_i(T,P)$ denotes the probability of finding a positive available radius within the i-th bin, its normalized weight in the positive available-radius distribution was calculated as:

$$\omega_i(T,P) = \frac{q_i(T,P)}{\sum_j q_j(T,P)} \tag{S16}$$

where the summation is taken over all bins with positive available radii. The state-dependent mean available cavity radius was then calculated as:

$$\bar{R}_{void}(T,P)=\sum_i \omega_i(T,P) r_i \qquad \text{(S17)}$$

The state-dependent mean-radius cavity-formation free energy, denoted as $\Delta G_{cav}^{\bar{R}}$, was subsequently evaluated according to:

$$\Delta G_{cav}^{\bar{R}}(T,P)=-k_B T \ln P\left[R\ge \bar{R}_{void}(T,P)\right] \qquad \text{(S18)}$$

Here, the cumulative probability was evaluated using all random probe points at the corresponding thermodynamic state. Because $\bar{R}_{\text{void}}$ does not generally coincide with the center of a discrete radius bin, the cumulative probability at $\bar{R}_{\text{void}}$ was obtained by linear interpolation between the neighboring radius-grid points. This approach directly relates the cavity size used in the free-energy calculation to the state-dependent void structure of water.

The corresponding cavity thermal response coefficient was calculated along each isobar as:

$$\chi_{cav}=-\left(\frac{d\Delta G_{cav}^{\bar{R}}}{dT}\right)_P \qquad \text{(S19)}$$

Because $\bar{R}_{\text{void}}(T,P)$ varies with temperature, Eq. S19 represents a total derivative along the state-dependent mean-radius path rather than a partial derivative evaluated at a fixed cavity radius. For each isobar, the $\Delta G_{cav}^{\bar{R}}$ data were interpolated at 1 K intervals using cubic spline interpolation, and χ_{cav} was obtained from the negative temperature derivative of the interpolated curve. The temperature at which χ_{cav} reached its maximum was identified as the cavity-response peak temperature.

The positive available-radius distribution was evaluated up to R_{max} = 12 Å using a bin width of 0.06 Å. The probability of observing R >12 Å was monitored explicitly and remained below 1.0×10^{-7} over the entire investigated thermodynamic range, indicating that the selected radius range captures essentially the complete positive available-radius distribution.

Figure S3 shows the isobaric evolution of $\bar{R}_{\text{void}}$. Along each isobar, the mean available cavity radius increases with temperature, reflecting the growth of accessible void space as the density decreases and molecular packing becomes less compact.[16,17] At lower pressures, this increase occurs sharply over a relatively narrow temperature interval, whereas increasing pressure shifts the variation toward higher temperatures and distributes it over a broader temperature range. The systematic dependence of $\bar{R}_{\text{void}}$ on both temperature and pressure confirms that the characteristic cavity size used in the present analysis is thermodynamic-state dependent.

To further demonstrate that the state dependence of $\bar{R}_{\mathrm{void}}$ arises from the systematic evolution of the full local void distribution rather than merely from a change in a single mean value, representative conditional positive-radius distributions, $p(R \mid R > 0)$, at temperatures 50 K below, at, and 50 K above the corresponding cavity-response peak temperature are shown in Figure S4. Along both the 200 and 400 bar isobars, increasing temperature shifts the distributions toward larger available radii, reduces the peak height, and extends the distributions toward the large-radius region. These changes indicate that both the typical local void size and the range of accessible free-volume environments increase during isobaric heating. The redistribution is more pronounced at 200 bar, where the isobar passes closer to the critical region, whereas the evolution at 400 bar is comparatively gradual. These results confirm that the full positive available-radius distribution changes substantially with thermodynamic state, providing direct support for evaluating ΔG_{cav} using the state-dependent $\bar{R}_{\mathrm{void}}(T, P)$ rather than a single fixed cavity radius.

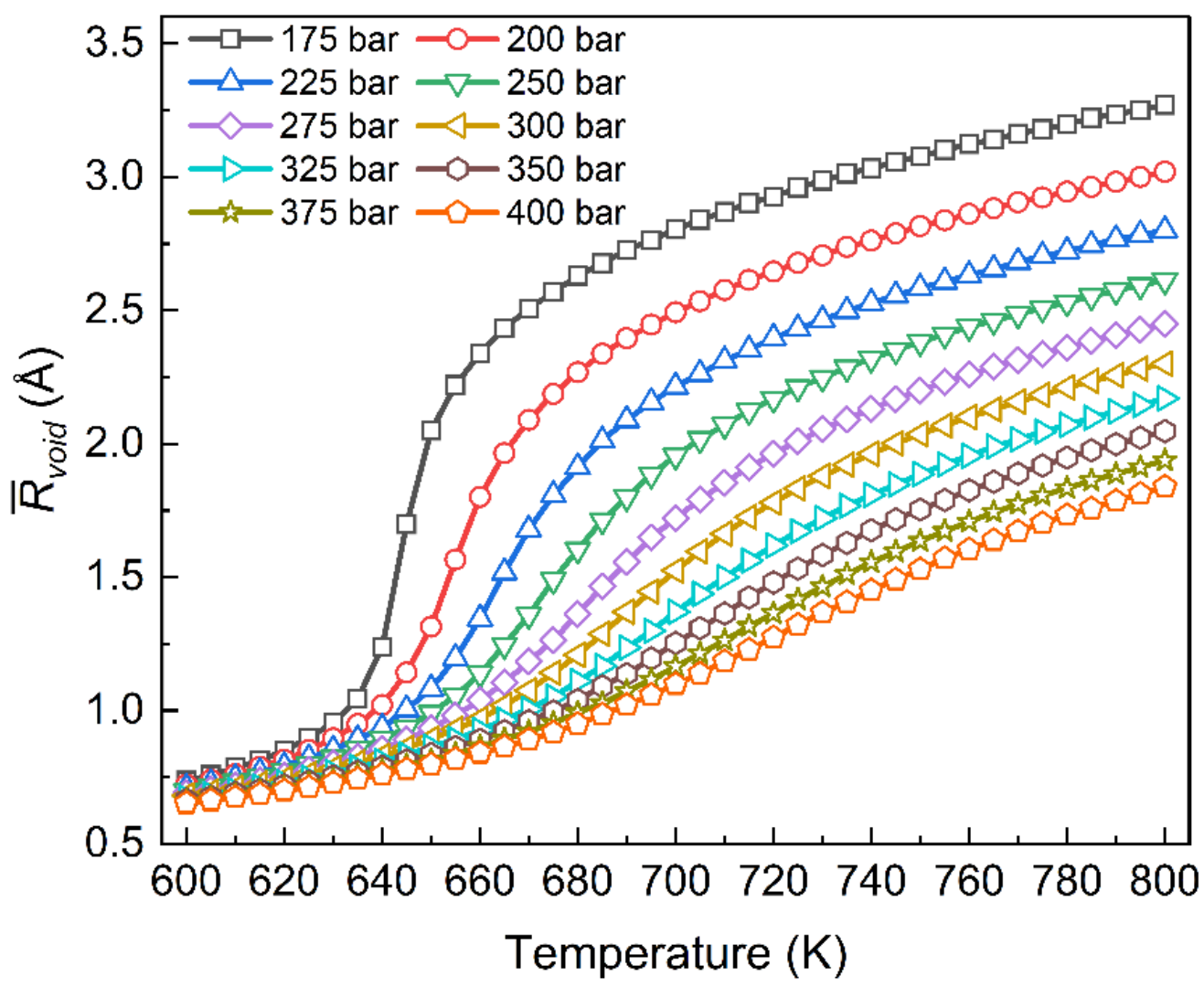

Figure S3. Isobaric evolution of the state-dependent mean available cavity radius, $\bar{R}_{\text{void}}$, in SCW. The mean radius was calculated from the positive available-radius distribution at each thermodynamic state according to Eq. S17.

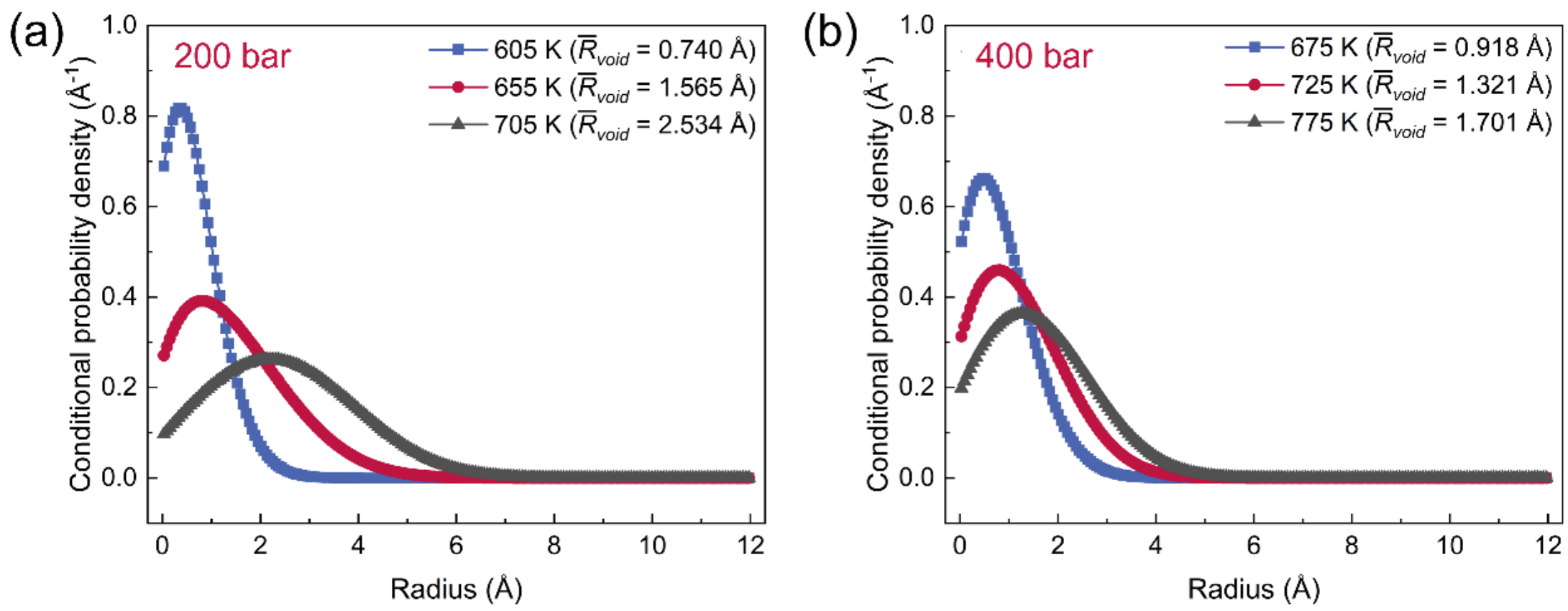

Figure S4. Representative conditional probability distributions of positive available cavity radii, $p(R \mid R > 0)$, along the (a) 200 bar and (b) 400 bar isobars. For each isobar, the distributions are shown at temperatures 50 K below, at, and 50 K above the corresponding cavity-response peak temperature. The state-dependent mean available cavity radius, $\bar{R}_{\text{void}}$, is indicated in the legend. The distributions were normalized over the positive-radius range such that $\int_0^\infty p(R \mid R > 0)\, dR = 1$.

Section S5: Approximate quantitative comparison between the χ_{CED} response ridge and the Widom-delta boundaries.

To provide an approximate quantitative comparison between the χ_{CED}-defined low-temperature boundary of the thermodynamic Widom region identified in this work and the structurally defined Widom delta,[18–22] the χ_{CED} maximum temperatures were compared with the percolation-threshold boundaries of the Widom delta over the investigated pressure range. The comparison was performed using reduced coordinates, $P_r = P/P_c$ and $T_r = T/T_c$, since the χ_{CED} response ridge and the Widom-delta boundaries were obtained from different thermodynamic frameworks. Specifically, the reduced pressure, $P_{r,\chi CED}$, and the reduced χ_{CED} maximum temperature, $T_{r,\chi CED}$, in the present work were calculated using the critical parameters of the TIP4P/2005 model adopted in this study, T_c = 640 K and P_c = 146 bar. In contrast, the Widom-delta boundary temperatures were obtained from the reduced-coordinate representation reported by Yoon et al.,[20] in which the structural percolation boundaries were nondimensionalized using the critical parameters of their corresponding-state framework. Therefore, the present comparison should be interpreted as an approximate corresponding-state validation rather than a strict point-by-point superposition in absolute P–T space.

The lower- and upper-temperature boundaries of the Widom delta were estimated from the reported gas-like and liquid-like percolation thresholds, respectively, and the corresponding reduced boundary temperatures were obtained by linear interpolation of the reduced P–T–Π_{gas} data reported for water.[20] As summarized in Table S1, the χ_{CED} maximum trajectory lies close to, but slightly below, the lower-temperature percolation boundary of the Widom delta over the investigated pressure range. The offset increases gradually with pressure, from $\Delta T_r \approx -0.015$ at 175 bar to $\Delta T_r \approx -0.071$ at 400 bar. This result indicates that the cohesive-energy response ridge is closely associated with the low-temperature flank of the structural Widom delta, although it is not strictly located inside the percolation-defined Widom-delta interval. This comparison supports the complementarity between the two descriptions: the Widom delta characterizes the structural crossover based on geometric percolation, whereas the χ_{CED} response ridge identifies the thermodynamic state at which solvent-network cohesion attenuates most rapidly along the isobaric heating path.

Table S1. Approximate quantitative comparison between the χ_{CED} maximum temperatures and the Widom-delta percolation-threshold boundaries over the investigated pressure range

P / bar	$P_{r,\chi CED}$	$T_{\chi CED}$ / K	$T_{r,\chi CED}$	$T_{r,delta,lower}$	$T_{r,delta,upper}$	$\Delta T_r = T_{r,\chi CED} - T_{r,delta,lower}$
175	1.199	644	1.006	1.021	1.045	−0.015
200	1.370	654	1.022	1.038	1.074	−0.016
225	1.541	662	1.034	1.056	1.103	−0.022
250	1.712	670	1.047	1.073	1.131	−0.026
275	1.884	679	1.061	1.090	1.158	−0.029
300	2.055	684	1.069	1.108	1.184	−0.039
325	2.226	692	1.081	1.125	1.210	−0.044
350	2.397	697	1.089	1.143	1.235	−0.054
375	2.568	702	1.097	1.160	1.261	−0.063
400	2.740	708	1.106	1.177	1.286	−0.071

Note: The lower and upper Widom-delta boundaries correspond to Π_{gas} = 0.09163 and Π_{liq} = 0.1489, respectively. Since Π_{liq} = 1 - Π_{gas}, the upper boundary is equivalently represented by Π_{gas} = 0.8511. Reduced quantities are reported to three decimal places.

SI References

(1) Thompson, A. P.; Aktulga, H. M.; Berger, R.; Bolintineanu, D. S.; Brown, W. M.; Crozier, P. S.; in ’t Veld, P. J.; Kohlmeyer, A.; Moore, S. G.; Nguyen, T. D.; Shan, R.; Stevens, M. J.; Tranchida, J.; Trott, C.; Plimpton, S. J. LAMMPS - a Flexible Simulation Tool for Particle-Based Materials Modeling at the Atomic, Meso, and Continuum Scales. *Comput. Phys. Commun.* **2022**, *271*, 108171.

(2) Abascal, J. L. F.; Vega, C. A General Purpose Model for the Condensed Phases of Water: TIP4P/2005. *J. Chem. Phys.* **2005**, *123* (23), 234505.

(3) Gallo, P.; Corradini, D.; Rovere, M. Widom Line and Dynamical Crossovers as Routes to Understand Supercritical Water. *Nat. Commun.* **2014**, *5* (1), 5806.

(4) Merchiori, S.; Le Donne, A.; Littlefair, J. D.; Lowe, A. R.; Yu, J.-J.; Wu, X.-D.; Li, M.; Li, D.; Geppert-Rybczyńska, M.; Scheller, L.; Trump, B. A.; Yakovenko, A. A.; Zajdel, P.; Chorążewski, M.; Grosu, Y.; Meloni, S. Mild-Temperature Supercritical Water Confined in Hydrophobic Metal–Organic Frameworks. *J. Am. Chem. Soc.* **2024**, *146* (19), 13236–13246.

(5) Corradini, D.; Rovere, M.; Gallo, P. The Widom Line and Dynamical Crossover in Supercritical Water: Popular Water Models versus Experiments. *J. Chem. Phys.* **2015**, *143* (11), 114502.

(6) Hockney, R. W.; Eastwood, J. W. *Computer Simulation Using Particles*; CRC Press: Boca Raton, 2021.

(7) Ryckaert, J.-P.; Ciccotti, G.; Berendsen, H. J. C. Numerical Integration of the Cartesian Equations of Motion of a System with Constraints: Molecular Dynamics of *n*-Alkanes. *J. Comput. Phys.* **1977**, *23* (3), 327–341.

(8) Huber, M. L.; Lemmon, E. W.; Bell, I. H.; McLinden, M. O. The NIST REFPROP Database for Highly Accurate Properties of Industrially Important Fluids. *Ind. Eng. Chem. Res.* **2022**, *61* (42), 15449–15472.

(9) Wagner, W.; Pruß, A. The IAPWS Formulation 1995 for the Thermodynamic Properties of Ordinary Water Substance for General and Scientific Use. *J. Phys. Chem. Ref. Data* **2002**, *31* (2), 387–535.

(10) Callen, H. B. *Thermodynamics and an Introduction to Thermostatistics*, 2nd ed.; John Wiley & Sons: New York, 1985.

(11) Marcus, Y. Internal Pressure of Liquids and Solutions. *Chem. Rev.* **2013**, *113* (8), 6536–6551.

(12) Dack, M. R. J. The Importance of Solvent Internal Pressure and Cohesion to Solution Phenomena. *Chem. Soc. Rev.* **1975**, *4* (2), 211–229.

(13) Roth, M. Cohesive Energy Densities Versus Internal Pressures of Near and Supercritical Fluids. *Molecules* **2019**, *24* (5), 961.

(14) Marcus, Y. The Structure of Mixtures of Water and Acetone Derived from Their Cohesive Energy Densities and Internal Pressures. *J. Mol. Liq.* **2020**, *320*, 112801.

(15) Lee, B.; Richards, F. M. The Interpretation of Protein Structures: Estimation of Static Accessibility. *J. Mol. Biol.* **1971**, *55* (3), 379-IN4.

(16) Kalinichev, A. G.; Churakov, S. V. Size and Topology of Molecular Clusters in Supercritical Water: A Molecular Dynamics Simulation. *Chem. Phys. Lett.* **1999**, *302* (5), 411–417.

(17) Choudhary, A.; Kundu, A.; Singh, C.; Sharma, A.; Pant, K. K.; Chandra, A. Supercritical Water: Density-Independent Angular Jumps. *J. Phys. Chem. B* **2025**, *129* (1), 329–337.

(18) Yoon, T. J.; Ha, M. Y.; Lee, W. B.; Lee, Y.-W. Probabilistic Characterization of the Widom Delta in Supercritical Region. *J. Chem. Phys.* **2018**, *149* (1), 014502.

(19) Ha, M. Y.; Yoon, T. J.; Tlusty, T.; Jho, Y.; Lee, W. B. Widom Delta of Supercritical Gas–Liquid Coexistence. *J. Phys. Chem. Lett.* **2018**, *9* (7), 1734–1738.

(20) Yoon, T. J.; Ha, M. Y.; Lee, W. B.; Lee, Y.-W. A Corresponding-State Framework for the Structural Transition of Supercritical Fluids across the Widom Delta. *J. Chem. Phys.* **2019**, *150* (15), 154503.

(21) Ha, M. Y.; Yoon, T. J.; Tlusty, T.; Jho, Y.; Lee, W. B. Universality, Scaling, and Collapse in Supercritical Fluids. *J. Phys. Chem. Lett.* **2020**, *11* (2), 451–455.

(22) Yoon, T. J.; Ha, M. Y.; Lee, W. B.; Lee, Y.-W. Monte Carlo Simulations on the Local Density Inhomogeneities of Sub- and Supercritical Carbon Dioxide: Statistical Analysis Based on the Voronoi Tessellation. *J. Supercrit. Fluids* **2017**, *119*, 36–43.
